# Multiplex protein profiling method for extracellular vesicle protein detection

**DOI:** 10.1038/s41598-021-92012-6

**Published:** 2021-06-14

**Authors:** Li Sun, David G. Meckes

**Affiliations:** Department of Biomedical Sciences, Florida State University College of Medicine, 1115 W. Call Street, Tallahassee, FL 32306-4300 USA

**Keywords:** Biological techniques, Cancer, Cell biology, Biomarkers

## Abstract

Extracellular vesicles (EVs) are small nanometer-sized membrane sacs secreted into biological fluids by all cells. EVs encapsulate proteins, RNAs and metabolites from its origin cell and play important roles in intercellular communication events. Over the past decade, EVs have become a new emerging source for cancer diagnostics. One of the challenges in the study of EVs and there utility as diagnostic biomarkers is the amount of EVs needed for traditional protein analysis methods. Here, we present a new immuno-PCR method that takes advantage of commercially available TotalSeq antibodies containing DNA conjugated oligos to identify immobilized protein analysts using real-time qPCR. Using this method, we demonstrate that multiple EV surface proteins can be profiled simultaneously with high sensitivity and specificity. This approach was also successfully applied to similar protocol using cell and serum samples. We further described the development of a micro-size exclusion chromatography method, where we were able to detect EV surface proteins with as little as 10 μL of human serum when combined with immuno-PCR. Overall, these results show that the immuno-PCR method results in rapid detection of multiple EV markers from small sample volumes in a single tube.

## Introduction

The idea of Immuno-PCR started from Sano and Canter’s work on a chimera of streptavidin and IgG binding domain which provide a bridge between the antibody and biotinylated DNA oligos in the early 1990s^1^. The very next year, Sano et al. published an application of the fusion product and termed it immuno-polymerase chain reaction (immuno-PCR)^2^. In this antigen detection system, antibodies conjugated by the chimera were used in ELISA-like procedure to detect immobilized bovine serum albumin (BSA) on plate, and then the DNA was amplified by PCR. The detection limit was pushed to about 600 molecules, which is 10^5^ more sensitive than ELISA. Since this early foundational work, these techniques have gained a lot of attention and been applied to biomolecular detection, such as cytokines, cell surface proteins and antigens from viruses and bacteria^3–7^. The next great leap forward of immuno-PCR happened 10 years after it was invented. In 2002, Simon Fredriksson and his colleagues developed the proximal ligation assay (PLA) for cytokine detection^8,9^. The group utilized large-scale SNP analysis with molecular inversion probes developed by Hardenbol et al.^10^. The PLA utilizes two DNA aptamer-based proximity antibodies, each containing a sequence binding to a short DNA connector. When the antibody binds pairwise to the target protein, the ligation could be performed between two tags when brought in close proximity. Then, the unique DNA products are amplified by qPCR for quantitative evaluation. The PLA could detect PDGF in zeptomole level without washes or separation and therefore outperforming the traditional immune-PCR with higher signal-to-noise ratios and no immobilization needed. Now, PLA approach is exploited in commercial applications available both in the form of pre-conjugated kits (Life Technologies, Carlsbad, CA, Olink Bioscience, Uppsala, Sweden) and high sensitivity protein immunoassay (Life Technologies ProQuantum).

Another advantage of immune-PCR is that the DNA tag could provide more flexibility compared to antibodies alone. Shibasaki et al. introduced a modified immune-PCR method named the MUSTag assay in 2009^11,12^. With this technology, antibodies linked to 100–300 bp long oligonucleotide could detect several important biomarkers. After a similar ELISA procedure, oligos were cut by EcoRI and quantified by Taqman qPCR. The different oligo-tags allows for simultaneous detection of multiple proteins with extremely high sensitivity of more than 10 femtogram. However, there are also other ways to fulfill the task of multiplex, like by mass spectrometry^13^ or sequencing^14,15^. Combined with single cell sequencing, the DNA oligo conjugated antibody method will simultaneously measure transcripts and surface proteins on a single cell level.

Extracellular vesicles (EVs) are cell-derived membrane sacs that range in size from 50 nm to over 1 um. EVs are distinguished by the site of biogenesis within the cell. Typically, larger EVs bud off from the cell surface and are termed microvesicles, whereas exosomes are generally accepted as vesicles that originate following budding and fusion into internal endosomal-derived multivesicular bodies (MVB). Exosomes are released from the cell following membrane fusion of the MVB with the plasma membrane. Exosomes and other EVs contain biologically active molecular cargos that mediate intercellular communication. EVs contain molecular information of their progenitor cells and are abundantly present in biological fluids. The unique properties of EVs have intensified efforts to discover EV biomarkers to diagnose or monitor disease^16–19^.

Ultracentrifugation, precipitation and size exclusion chromatography (SEC) are the most widely used methods in the EV isolation field. In past decade, several advanced and well-developed techniques have been adopted into the EV field for both separation and characterization, including microfluidics, chip, advanced microscopy and flow cytometry^20–22^. However, very few studies describe the use of immuno-PCR method in the EV field. Recently, Di Wu et al. developed a PLA based protein profiling assay for single exosome analyses^23^. The proximity-dependent barcoding assay (PBA) utilizes single-stranded DNA clusters to barcode individual exosomes. Using this method, the group has successfully profiled 38 different surface proteins simultaneously on individual exosomes.

In this study, we developed and optimized a protocol based on commercially available TotalSeq-A antibody (BioLegend Inc.) which is the key component in the CITE-seq method for single cell surface protein profiling^14^. In our protocol, after binding the TotalSeq-A antibody pool to the protein sample blotted on the small nitrocellulose (NC) strip, an adapter with 3′ oligo-dT is annealed to the TotalSeq antibody tail and filled by DNA polymerase at room temperature. Protein specific upper primers were designed on the barcode region close to the 5′ of end of the oligo on TotalSeq-A, and the universal downstream primer binding sites were designed on the adapter. Each antibody signal could be amplified with the primer pairs on any real-time PCR machine. Combining this approach with micro-SEC, fractions from as little as 10 μL serum sample could be used to profile EV biomarkers. The TotalSeq-A antibody could also be used to successfully profile cell surface proteins, greatly enhancing the utility of this method. Overall, we developed a fast, sensitive and cost-effective multiplexing protein profiling assay that may serve as an ideal platform for EV biomarker studies and diagnostics.

## Results

### TotalSeq antibody assay can profile proteins from cell lysates and EV samples

**Figure 1:**
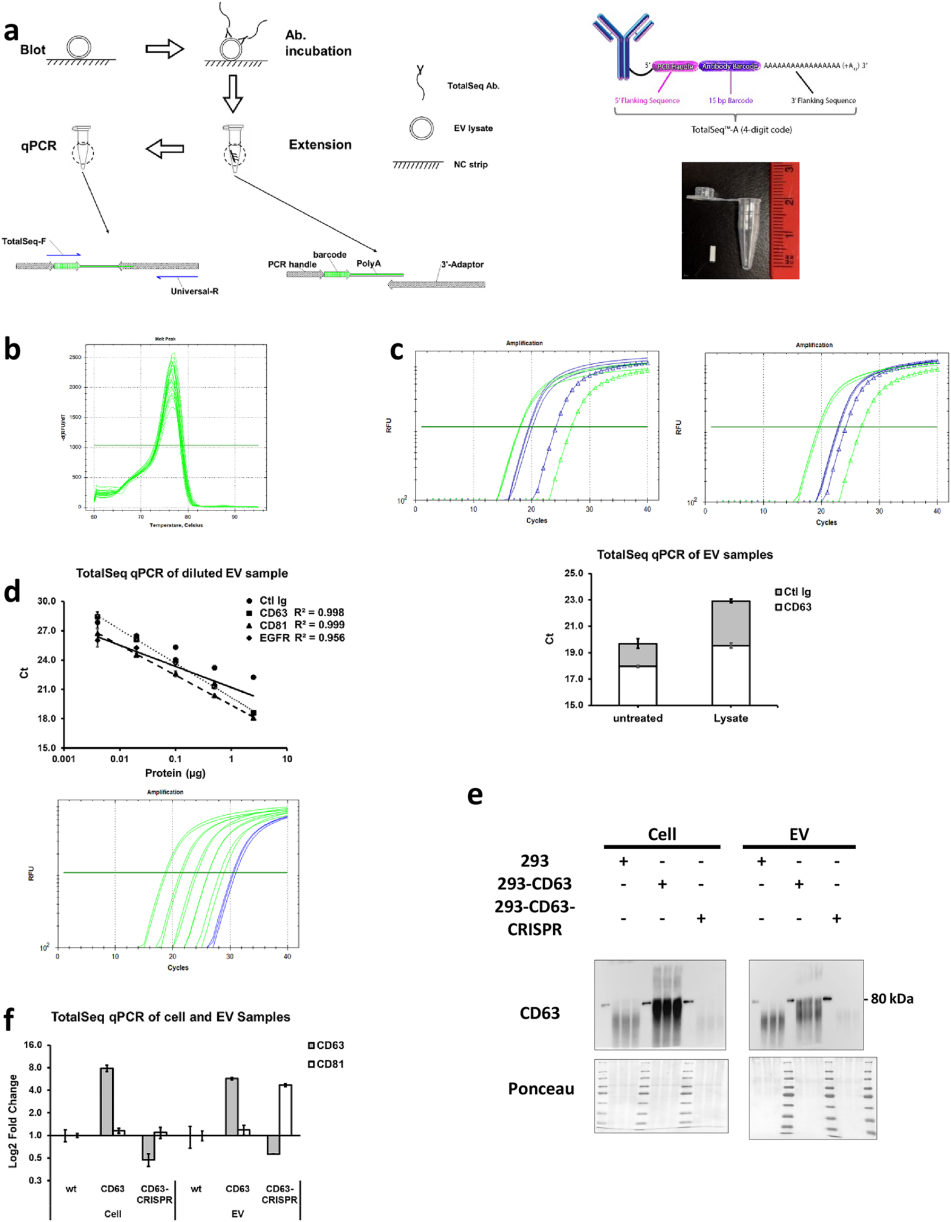
Protein sample were profiled with TotalSeq antibody. (**a**) Basic work flow for detection of immobilized protein sample by TotalSeq-A antibody. Structure of conjugated oligo in TotalSeq-A antibody (up right), reprint from BioLegend website with permission. Size and comparison of pre-cut strip and PCR tube (bottom right). (**b**) Melting curve of different TotalSeq antibodies on EV protein samples in triplicate. (**c**) Intact (upper left) or lysised (upper right) #1 EV samples were tested with TotalSeq CD63 (green) and control mIgG (blue) antibody in Triplicate. Equal amount of BSA (triangle) was used as negative control. Ct values of CD63 (white) and control mouse IgG (gray) are overlaid in column chart to show the difference. (**d**) Serial dilution of #1 EV sample was incubated with TotalSeq antibody CD63 (square), CD81 (triangle), EGFR (diamond) and control IgG (circle). Amplification curve (bottom) of CD63 on EV dilution (green) and BSA (blue). All experiments were performed in duplicate. (**e**) CD63 immunoblot result of EV and whole cell lysate. Full-length blots are presented in Supplementary Figure S7. (**f**) TotalSeq qPCR result on the same samples as in (**e**). Data were normalized to wild type HEK293 samples.

To take advantage of commercially available antibody pool from BioLegend, we developed a working protocol around the sequence of the oligo conjugated. The oligos of TotalSeq antibody were designed by BioLegend in three formats to fit in different circumstances. TotalSeq-A is the only format with confirmed nucleotide sequence and a poly-A tail, the other two contain sites of random nucleotide incorporation which brings unpredictable results on the following extension and qPCR step. Therefore, we designed a DNA oligo fragment (named as 3′-adaptor) which contain poly-T region on 3′ end to fulfill annealing with 3′ poly-A tail on TotalSeq-A oligo and a primer binding site for a designed universal reverse primer (Fig. 1a).

In preliminary experiments, several chosen TotalSeq-A oligos were synthesized to test the feasibility of the entire protocol. Those oligos were either alone or in the mixture with other oligos to investigate specificity, robustness and dynamic range of this assay (Supplementary Fig. S1–2, Supplementary Table S1). First, synthesized DNA oligos were incubated with the 3′-adaptor and DNA polymerase to be converted into full length DNA template. Second, the reaction tube was heated to 95 °C on thermocycler to inactivate DNA polymerase. This step will also help release oligos from the antibody and strip when the protocol is applied to real protein samples and TotalSeq-A antibody. Third, the reaction containing extension products were used as a template in the following qPCR reaction with universal reverse primers and TotalSeq specific forward primer set. The melting curve showed a homogenies peak around 76–77 °C (Fig. 1b), which is the result of a poly-A-T region in the final product. No peak was observed in the reaction tube lacking either oligos, DNA polymerase or primers (Data not shown). Moreover, to increase the sensitivity of the assay, a universal forward primer which binds to the 5′ flanking sequence of all TotalSeq-A oligos could be combined with the universal reverse primer in first round PCR for 15 cycles. This product could be used as template for specific primer amplification in secondary round qPCR (Data not shown).

When analyzing a protein sample with this protocol, the first step is immobilization on a pre-cut NC membrane strip (Fig. 1a). After appropriate blocking buffer incubation, the TotalSeq-A antibody pool was added to the 1.5 mL Eppendorf tube containing the nitrocellulose strip commonly used in dot blot or western blot protocols. Unbound or non-specific antibody binding was removed following PBST wash after overnight incubation. Then, instead of using a secondary antibody as readout, the strip was transferred into a PCR tube to performs the extension and qPCR steps as describe in the methods.

Following the establishment of the protocol, protein samples (from cell and EVs, Supplementary Fig. S3) were tested with TotalSeq-A CD63/CD81/EGFR/isotype control antibody pool. Same amounts of BSA were blotted on the strip as negative control to show the specificity of antibodies and background signal. Ct values of all the antibodies in cell or EV lysate samples were much lower than in BSA, indicating a positive signal on the sample strips (Fig. 1c). The mouse IgG1-κ isotype control TotalSeq-A antibody was always used with same concentration to show the noise level or unspecific binding of antibody to strip in all the experiments described. The Ct value from any antibody was accepted as real signal only when the ΔCt value between the antibody and isotype control was great than one. Different blocking reagents and conditions were extensively tested, but the background level of control mouse IgG still amplified with Ct around 25 to 30 (Data not shown). The EVs were lysed with 0.1% SDS as recommended in the protocol because it increased the signal to noise ratio. As see in the test of CD63 and control Ig, Ct value of CD63 is increased from 18 to 19 on lysed sample, but ΔCt is also increased from 1.8 to 3.0 (Fig. 1c).

Dynamic range of the assay on EV sample is about 0.05 to 5.0 µg of total protein (2-logs) (Fig. 1d), which is similar to other antibody-based protein quantification methods like western blot. So, all the samples in the following experiments were used 0.5–1.0 µg based on the 660 nm Protein Assay results. From the EV dilution curve, the results showed that the more protein used in the assay, the higher signal to noise ratio (Fig. 1d). To further compare our method with traditional protein quantification method, we used HEK293 cell line stable transfected or knockout CD63 molecule by CRISPR-Cas9 system. Cell or EV samples were quantified and compared side by side with western blot and TotalSeq-A antibody assays, and results showed that the two approaches were comparable (Fig. 1e,f). However, the TotalSeq method is time and sample saving, and has a greater potential for multiplex detection of different target from one same sample (Table 1).

**Table 1:** Comparison of Western blot and TotalSeq assay.

	Western blot		TotalSeq assay	
Sample amount	10 µg		0.5 µg	
Antibody used	First antibody (2 mL, 1:1000)		TotalSeq antibody (100 µL, 1:2000)	
	Secondary antibody (2 mL, 1:5000)			
Timing	Gel running	2 h		
	Transferring	2 h	Blocking	1 h
	Blocking	1 h	Antibody	2 h to o/n
	First antibody	o/n	Wash	0.5 h
	Secondary antibody	1 h	Extension	0.1 h
	Wash	1 h	qPCR	1.5 h
	Imaging	0.5 h		
Multiplex	+*		+++	

*When using fluorescent secondary antibody or stripping membrane.

### TotalSeq antibody assay could be used on cells under similar protocol

**Figure 2:**
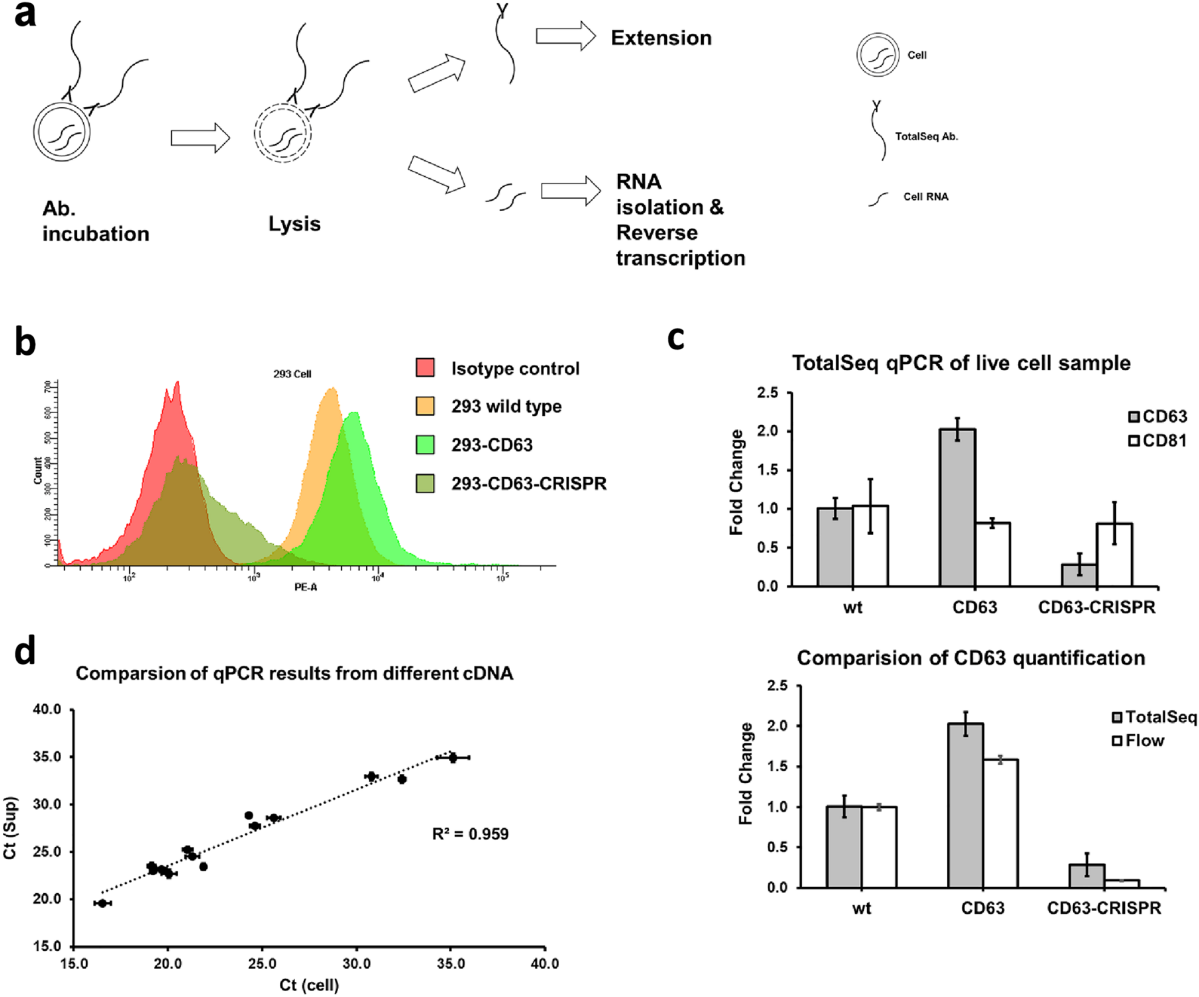
Cell surface protein were profiled with TotalSeq antibody. (**a**) Workflow for cell samples. (**b**) Overlaid histogram plot flow cytometry results of 293-WT, 293-CD63 and 293-CD63-CRISPR cell stain with either CD63-PE or isotype control-PE antibody. Representative result of three independent samples shown. (**c**) Result on the same cell sample as in (**b**) with TotalSeq qPCR method. Data were normalized to wild type sample. (**d**) Gene expression level was tested from RNA isolated by Trizol from cell (X axis) or cleared supernatant (Y axis). Samples were showed in triplicate.

The TotalSeq antibody has been successfully used in single cell sequencing to acquire transcriptome and surface proteomic data simultaneously^14^. One of the critical points of their method is to use high quality flow cytometry grade antibodies. So, we also test our protocol on the cell level, with minor modification from CITE-seq staining protocol suggested by BioLegend (Fig. 2a). After staining with TotalSeq antibody, cells were lysed by freeze–thaw cycles on dry ice and then boiled in a thermocycler. Cleared supernatant containing TotalSeq antibody oligos was then used in extension reaction as in the protocol for protein sample.

HEK293 cell lines with overexpressed CD63 or CD63-CRISPR were stained with either TotalSeq antibody or PE-CD63 antibody and compared directly to flow cytometry. The results were comparable with the data from the traditional flow cytometry method for cell surface staining (Fig. 2b,c). Moreover, this cell lysate before extension step also contains RNAs released from cell plasma, which could be used to obtain transcriptome data from the same cell sample. Total RNA from either cell or cleared supernatant were isolated by Trizol and converted to cDNA. We used mRNA expression levels of 16 genes to evaluate cDNA quality from these two different sources by qPCR (Fig. 2d, Supplementary Fig. S4), which include both high expressed housekeeping genes and low expressed integrin genes. RNA from cleared supernatant is highly corelated with RNA from the whole cell (R^2^ = 0.959), but Ct value is higher (4.7 ± 1.3) in all the genes tested, even if same amount of total RNA had been used for reverse transcription reaction (Supplementary Fig. S4). Using the cDNA from cell lysates or the cells directly, Ct value from TotalSeq antibody assay could also be normalized to GAPDH level with ΔΔCt method on one qPCR plate.

In some previous publications^24^, cell lysate from detergent buffer or even water could be directly used for reverse transcription without the RNA isolation step. There is even commercial product available (iScript RT-qPCR Sample Preparation Reagent, Bio-Rad, 1708898) that can be used for this purpose. Therefore, we also tested direct reverse transcription on our cleared supernatant sample. The results showed that cDNA directly from cleared supernatant has more complicated pattern compared with RNA from cell or lysate, but the Ct value is much higher than the other sample conditions (Supplementary Fig. S4). Therefore, for protocol simplification and normalization purposes, cleared supernatant could be used both in the TotalSeq assay extension reaction and direct reverse transcription. Using this approach, the extension product and cDNA can be run on the same qPCR plate for protein expression and GAPDH levels at the same time. But other genes (like CD63) need to be fully compared with Trizol isolated RNA to prove correlation before using this simplified protocol.

### Micro-SEC column is a fast and high purity EV isolation method from small volume serum samples

SEC is one of the popular EV isolation methods because of several extraordinary features^25^. SEC does not reply on other expensive instruments and can be packed in any laboratory with only matrix and empty column (even in syringe)^26^. The main purpose of the column is to collect fractions by gravity flow. After the purification of each sample, the column could be regenerated with clean-in-place buffer or just simply discard. One of the major advantages of SEC is that it maintains high EV purity and maintains most of their biological activity, as no other chemicals or hash purification conditions (i.e., ultracentrifugation) are needed.

**Figure 3:**
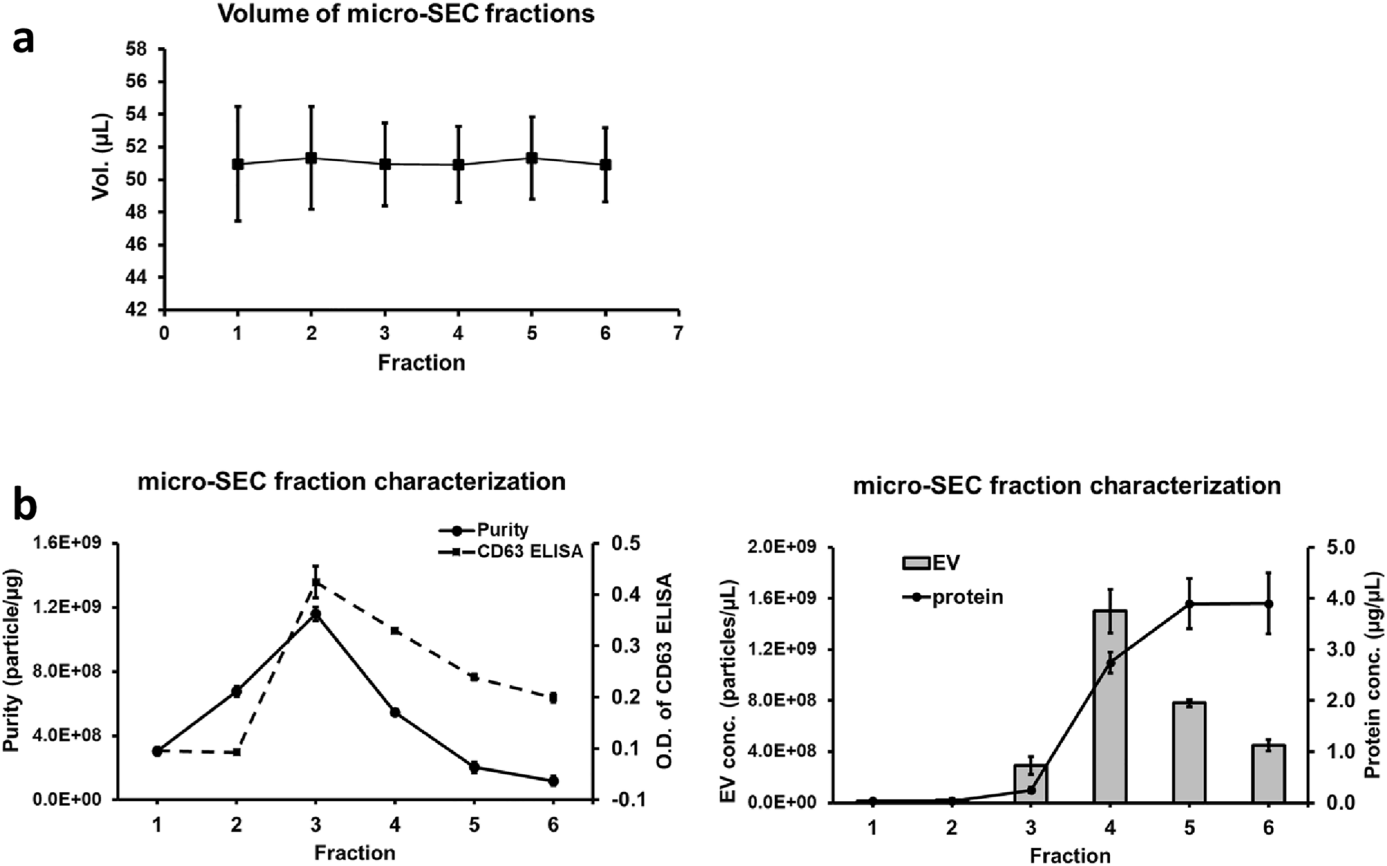
Micro-SEC method was developed for rapid serum EV isolation. (**a**) 25 uL pre-cleared and diluted serum sample was loaded on micro-SEC column and fractions were collected. (**b**) EV concentration was measured by NTA and protein content were assayed by OD660 (left). The purity of EV samples was calculated by particle/µg protein. CD63 Direct ELISA was used to measure the EV peak in all the fractions. All the data were collected from two individual columns.

To apply the TotalSeq assay to small volumes of clinical samples, a micro-size exclusion method was developed similar to larger SEC (10 mL bed volume) packed columns. Matrix was packed into a spin column (Fig. 3a) and fractions could only be collected by drop which is the smallest achievable volume by gravity flow. By measuring the weight of each drop, the volume is around 50 µL and consists of the first 6 fractions (Fig. 3a). Twenty-five microliter of pre-cleared serum sample was used to test the performance of micro-size column. NTA and protein quantification results showed that micro-SEC has similar enrichment capacity and purity compared with larger column SEC (Table 2). The EV marker CD63 was tested by direct ELISA method with about 30 µL fraction directly coating on the plate. The EV with the highest purity was enriched in fraction 3 with about 30% recovery and soluble proteins were eluted in the later fraction 4 or 5 (Fig. 3b). Smaller sample volumes as low as 10 µL were also tested. The purity and CD63 peak shifted from fraction 3 to fraction 4 when 10 uL of sample were run through the column (Data not shown). As the fraction volume contains 50 µL, 25 µL loading volume will have lower dilution factor (1:2) compared with 10 µL (1:5).

**Table 2:** Comparison of regular and micro-size SEC.

Sample	Mean (nm)	Mode (nm)	Purity (/µg)	Recovery
Serum	128.0 ± 19.8	103.5 ± 2.1	8.50E + 07	–
Regular SEC fraction 3	141.5 ± 10.6	100.0 ± 7.1	3.83E + 09	47.6%
Micro-SEC fraction 3	125.0 ± 17.0	112.5 ± 21.9	3.20E + 09	26.0%
Micro-SEC fraction 4	143.5 ± 3.5	118.0 ± 2.8	2.70E + 09	65.5%

Taking together, by using micro-SEC method, serum EVs could be purified in 5 min from as little as 10 µL serum sample, and directly used in downstream analyses like ELISA.

### Micro-SEC combined TotalSeq protocol provide a sensitive and fast method for EV protein profiling from serum sample

**Figure 4:**
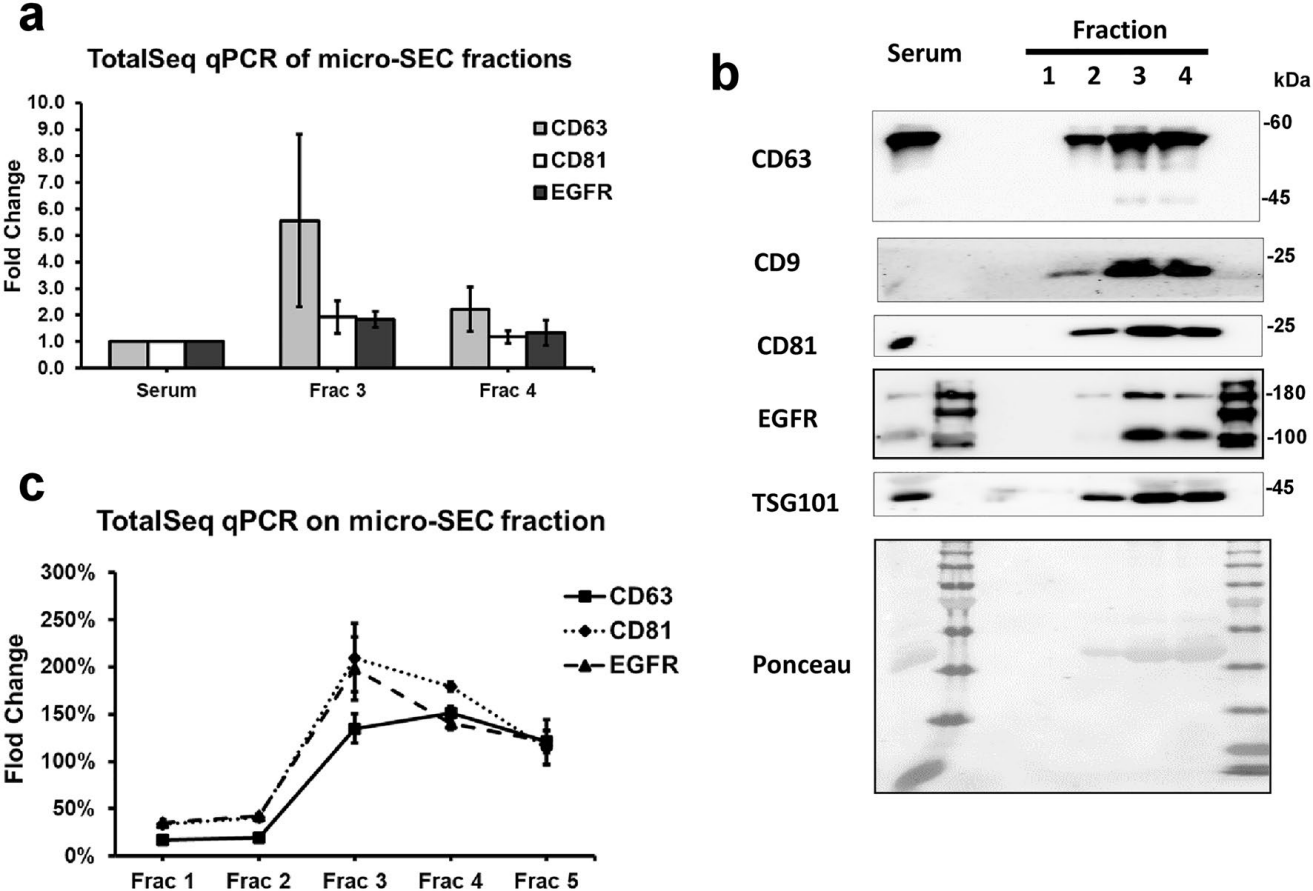
Serum EV protein was profiled using micro-SEC combined with TotalSeq antibody. (**a**) Fraction 3 and 4 collected from micro-SEC on human serum sample were tested with TotalSeq qPCR. Equal protein was blotted on the strip. Data were normalized to serum sample. (**b**) Equal volume of Fraction 1–4 from the micro-SEC were run on SDS-PAGE for immunoblot on EV markers. Full-length blots are presented in Supplementary Figure S8. (**c**) Same fraction sample as in (**b**) were analyzed by TotalSeq qPCR also blotted by equal volume. Data were normalized to serum sample.

Fraction 3 and 4 collected from micro-SEC column were analyzed by TotalSeq antibody and normalized to serum samples based on equal total protein (Fig. 4a). In some cases, serum alone did not produce any positive signals on TotalSeq assay qPCR. This is likely because the EV levels were too low to detect when only one microgram of serum protein was blotted on the strip. But after EV enrichment, CD63 and EGFR signals were much higher than background level (Data not shown). When using equal volume of micro-SEC fraction on TotalSeq assay, a similar peak of EV markers was show as western blot (Fig. 4b,c). Several EV isolation methods were performed to enrich EVs for analyses, including ultracentrifugation, ExtraPEG and micro-SEC as the method describe above. When all enriched EV samples were applied to TotalSeq antibody assay based on equal protein amount, we could clearly see that micro-SEC and ExtraPEG have similar pattern of enriched EV markers comparing with serum alone (Table 3). Taken together micro-SEC is a viable method for EV enrichment from small volumes of biological fluids comparable to other methods widely used in the field. However, based on ease of use and not needing expensive equipment micro-SEC may prove to be ideal in a clinical diagnostic setting.

**Table 3:** Comparison of micro-SEC with other EV enrichment method.

Sample	Dilution factor	Protein Conc. (µg/µL)	TotalSeq assay*		
			CD63 (%)	CD81 (%)	EGFR (%)
Serum	1	77.5	100	100	100
PEG pellet	1	9.8	602	285	162
Extra-PEG EV	1	2.0	994	238	183
UC EV	1	2.9	746	174	260
Micro-SEC fraction 3	2	0.8	925	158	150
Micro-SEC fraction 4	2	2.5	292	89	125

*Normalized to serum sample.

## Discussion

In this study, we developed a protocol around the TotalSeq-A antibody to profile membrane immobilized protein samples, even at the whole cell level (Figs. 1, 2). The idea was started from the large commercially available pool of TotalSeq antibody developed by BioLegend for sequencing purpose and the need to quantitatively detect multiple EV surface proteins from small sample volumes^14^. This method based off of the principle of immuno-PCR the qPCR quantification of resident oligo will reflect the amount of protein target. We also assumed that the poly-A on 3′ of TotalSeq oligo will be linked to poly-T oligo under enzyme activity of DNA polymerase. All of those reaction conditions and adaptor design were verified in experiments detailed in the supplemental figures. Seven different oligos were tested without interference of each other (Supplementary Fig. S1–2). We strongly suggest that investigators first test the oligo compatibility before applying any other TotalSeq-A antibody not tested here on our protocol. qPCR detection limit of oligos has reached femtomole level in most cases, and the primers for qPCR showed very high specificity (Supplementary Table S1). To further apply oligo conjugated antibody on protein tests, we did not choose the 96 well plate as immobilized surface as in conventional immuno-PCR^27–29^ mostly because of the coating efficiency. Instead, dot blot method was used to minimize consumption of relative expensive antibody (as little as 100 µL) and the use of limited EV samples (as little as 1 µL). Usually, dot blot image analysis needs to use signal on blotted sample substrate background level which either from edge of sample circle or an empty blot. However, we choose to pre-cut the membrane into rectangle strip with comparable size of 1 µL sample blot circle on large membrane. In this way, the signal from whole strip will only reflect the sample signal. Another advantage of the small strip is, it could be easily washed in common microfuge tube and transferred into PCR tube for further reaction. Considering the size of strip, in the antibody incubation step, as little as 30 µL of TotalSeq antibody solution is enough to cover the whole strip, but the reaction volume on such scale has not been tested. The final simulation was tested by using biotin label BSA and streptavidin conjugated oligo. 500 ng to 0.5 pg biotin-BSA were used as sample, and results showed 2-log dynamic range and 0.5 ng detection limit which was similar to the experiment using TotalSeq antibody (Fig. 1d), and the comparable performance on conversional dot blot assay (Supplementary Fig. S5–6).

By using the TotalSeq-A antibody on protein samples under the established protocol, we showed that the TotalSeq assay gives comparable data with current protein quantification methods, such as western blot, ELISA and flow cytometry (Figs. 1e,f, 2b,c). From commercially available antibody pools, we chose two representative exosome surface markers CD63 and CD81, and a functional cell surface molecule frequently found in EVs, EGFR^27,30–32^. In a previous paper the authors suggested to use the TotalSeq antibodies within 6 months^14^, because of the oligo conjugation stability with the antibody. Based on our test, TotalSeq antibody is also good for our assay with the store condition in 4 °C for at least 6 months.

Normalization also needs to be carefully considered. For its purpose, TotalSeq antibody was designed to profile surface proteins which may not suitable for normalization purposes because of protein turnover rate depending on cellular conditions^33^. However, there are still some potential candidates, like β2-microglobulin and HLAs that may serve this purpose due to low protein turnover rates. For EV studies, a lot of researchers use tetraspanins (CD63/CD81/CD9) to quantify exosomes, but there are still some studies showing that these tetraspanins will up- or down-regulated under pathological conditions^34–36^. Non-protein binding molecules like AnnexinV is also a good potential candidate to normalized protein content to phospholipid. In our experiment, total protein was used to normalize between different samples and show similar results in traditional western blot. Especially when EV samples are lysed with 0.1% SDS to obtain more consistent results. And for cell samples, we normalize TotalSeq antibody data to cytoplasmic GAPDH mRNA level as common qPCR reference gene. The use of cleared supernatant in mRNA expression experiments needs to be carefully considered. From our data on 16 genes, different patterns were observed compared with Trizol isolated RNA (Supplementary Fig. S4). Based on ΔCt compared with cell RNA set, all the 16 genes could be classified into 3 different groups. The first and second groups had similar expression patterns, but the first group had a much higher ΔCt value (11.6 vs. 4.6). In the first group, there were 9 genes of the total 16, including two housekeeping genes (GAPDH and ACTB). But in the third group, direct cDNA has lower Ct than the other two groups. This suggests for genes in this group, more sensitive detection will be obtained if directly using cleared supernatant for transcriptome analyses. Especially for the CHMP4C gene whose expression is too low to be detected in both isolated RNA samples.

Immuno-PCR and its advanced form “PLA” have proven great potential for protein profiling and quantification. The traditional immunoassay relays on enzyme or fluorophore linked antibodies to amplify signals from an immobilized surface. Therefore, its multiplexity capacity is limited by host of secondary antibody in western or ELISA assays, or fluorescent channel chosen to avoid cross-reactivity. When using DNA oligo as a barcode for each antibody, those oligos could be distinguished by highly specific primer design. Our results show that at least up to 7 different oligos could be profiled by unique primers and gives similar Ct to each primer pair analyzed independently. As in PLA, our protocol also has the advantage of low consumption of antibody and sample. Moreover, based on the principle of PLA, we assume that two-probe system could be used instead of our one-probe on-strip system to reduce background, skip multi-wash steps and increase the signal to noise ratio. But the linker in multiplex PLA needs to be carefully designed to increase ligation reaction’s specificity. There are already several successful applications described for the use of multiplex PLA on serum and in situ samples^37–39^.

To increase the detection limit of serum EV proteins, appropriate pretreatment and isolation steps are also critical points to be considered. SEC has been proved to be one of the fastest EV isolation methods from bodily fluid^25,40^, and that is why we developed a micro-size droplet-fractionated protocol on microliter scale serum sample. From our results, EVs from as little as 10 µL serum could be isolated in less than 5 min on an in home-made, reusable/deposable column, and seamlessly applied to our protein multiplex profiling system. EVs derived by tumor tissue and released in circulating system play an important role in cancer growth, invasion and metastasis^27,35,36^. These EVs will function in multi-aspects of cancer pathogenesis: oncogene mutations carried in DNA fragment could promote recipient cell proliferation^41^, transferring drug-resistant phenotype^42,43^, miRs inhibit tumor suppress genes^44,45^, surface MMPs remolding tumor microenvironment^46^, specific integrins target different cell type to promote metastasis^47^, transferring viral miRs or genome fragments^31,48^ and so on. Under pathological conditions, circulating exosomes may alter in total number, subpopulation ratio, surface protein marker and specific miRs^34–36,49^ which makes them perfect non-invasive specimens for cancer diagnostics, prognostics and even therapeutic purposes. Obviously, the screening of proteins, miRs and DNA altogether will greatly expend marker combination candidates and increase specificity and sensitivity^50,51^. Our method provides a possible resolution to test DNA, miRs and protein all in one single qPCR plate. cDNA reverse transcripted from miR, unique DNA/RNA fragment, extension product from TotalSeq protocol, or even DNA methylation assay could be run under a same qPCR protocol with suitable primer/probe design (Data not shown). Moreover, when the oligo standard is introduced into the qPCR reaction, absolute molecular quantification is also possible for this assay. Or the goal will be achieved by simply switching the qPCR step of our assay to droplet digital PCR, but have not be tested.

Overall, we provide a simple, cost-effective and highly flexible methods for protein profiling from various sample sources that will likely be of benefit to not only to researchers but also the clinical diagnostic setting.

## Methods

### Cell culture and stable cell line construction

HEK293 (ATCC, CRL-1573) and HEK293T cells (ATCC, CRL-11268) were cultured in Dulbecco’s Modified Eagle’s Medium (DMEM, Sigma, D5796) supplemented with 10% fetal bovine serum (FBS, ThermoFisher, 26140079) and penicillin–streptomycin (Corning, 30-002-CI). #1 cells (an Epstein-Barr virus (EBV) infected lymphoblastoid cell line) were cultured in RPMI-1640 medium (Sigma, R8758) supplemented with 10% FBS and penicillin–streptomycin. All cells were incubated at 37 °C in an atmosphere supplemented with 5% CO_2_.

HEK293 cells stably expressing CD63-GFP were generated by lentivirus transduction and puromycin selection using Exosome Cyto-Tracer, pCT-CD63-GFP (Systems Biosciences, CYTO120-PA-1) in our lab as previously described^52^. Briefly, medium containing lentivirus particles was harvested from HEK293T cells transfected with pCT-CD63-GFP and packaging plasmids pMD2.G (Addgene, #12259), pRSV-Rev (Addgene, #12253), and psPAX2 (Addgene, #12260, kind gifts from Dr. Didier Trono) at 48 h post-transfection. Medium was then centrifuged for 10 min at 1000 g and filtered through a 0.45 μM filter. HEK293 cells were incubated with the resulting lentivirus-containing medium and a 10 μg/mL final concentration of Polybrene (Sigma, H9268). Following 24 h of incubation, the medium was replaced with complete DMEM for 24 h and then puromycin was added at a final concentration of 2 μg/mL. Cells were subcultured for 3 weeks under puromycin selection to eliminate non-transduced cells.

### EV isolation and characterization from cell conditioned medium

EV-depleted FBS was prepared by centrifugation at 100,000 g for 20 h, then filtered through a 0.2 µm filter. Cells were first seeded in regular medium and then change to EV-depleted medium (contain 10% EV-depleted FBS) for 48 h before harvest. EVs from cell conditioned medium were isolated by ExtraPEG protocol as previous described^53^. Briefly, medium was first cleared by differential centrifuged at 500 g for 5 min, 2000 g for 15 min and 10,000 g for 30 min to remove cellular debris, apoptotic bodies and large vesicles. Then, cleared medium was mixed with PEG-6000 (Sigma, 81260) solution to 8% final concentration and incubated at 4 °C overnight. On the following day, crude EV were pelleted by centrifuged at 3000 g for 1 h. Then, pellet was resuspended in particle-free PBS (pass through 0.2 µm filter) and ultracentrifuged at 100,000 g for 70 min to wash contaminating protein and PEG. Purified EV were resuspended in particle-free PBS and store in − 80 °C until use.

Nanoparticle tracking analysis (NTA) was performed to calculate concentration and size by using the NanoSight LM10 (Malvern) with set the camera level to 13 and detection threshold to 5^53^.

Total protein level of EV was quantified by Pierce 660 nm Protein Assay (ThermoFisher, 22660) in present of 0.1% SDS to lysis EVs.

### EV isolation from human serum

De-identified human serum samples were purchased from BioIVT (Hicksville, New York). BioIVT certifies that all human samples were collected under IRB approved protocols. Thawed human serum aliquot was diluted with filtered PBS (1:1) and pre-cleared to remove debris by centrifugation at 10,000 g for 15 min. Then, EVs were isolated utilizing different methods. For ultracentrifugation, serum was further diluted with 1 mL particle-free PBS and centrifuge at 100,000 g on TLA-120.2 rotor for one hour. For ExtraPEG method, as described in previous paper^53^, serum was diluted and mixed with PEG solution (final concentration 8%) for 30 min. After a 3000 g centrifugation, the pellet was resuspended in PBS and further purified with 1-h ultracentrifugation at 100,000 g. All the ultracentrifuged pellets were resuspended to its original volume to make them comparable. Then the purified EVs from the different methods were tested by NTA, protein quantification, western blot or TotalSeq antibody assay in side-by-side comparisons.

### Protocol of using TotalSeq-A antibody for protein profiling

TotalSeq-A antibodies tested in this paper were purchased from BioLegend Inc. (San Diego, CA) and barcode information is shown in Table 4. All the DNA oligos and primers were synthesized from IDT Inc. (Coralville, Iowa). Forward qPCR primers (TotalSeq-F) were designed to bind on barcode region (shown as red in Table 4) of TotalSeq-A 5′-handle and match the Tm of universal reverse primer (Universal-R) designed on 3′-adapter. The full length 5′-handle oligos were synthesized only to verify and optimize protocol (Supplementary Fig. S1–2, Supplementary Table S1).

**Table 4:**
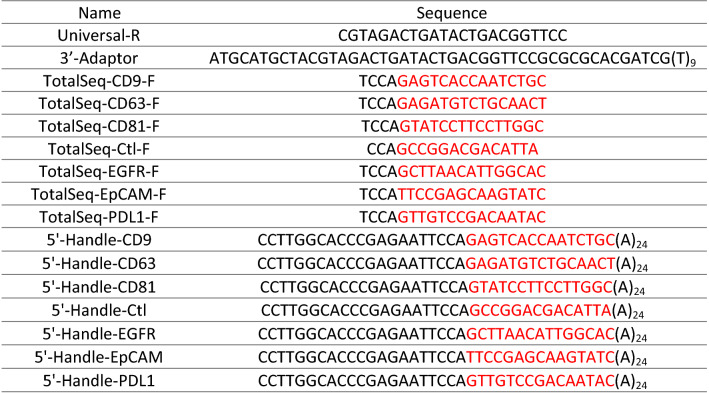
Sequence of TotalSeq primers and oligos.

*Barcode regions were shown in red.

Protein sample for the TotalSeq antibody assay could be from cell lysates in radioimmunoprecipitation assay buffer (RIPA) or EV sample lysed in 0.1% SDS. One microgram of protein sample from cell lysate or EVs was carefully blotted onto a pre-cut strip of nitrocellulose membrane (GE Healthcare, #10600002). After the strip had dried completely, one µL of casein blocking buffer (2.5% casein in PBS, 0.05% tween-20, 100 µg/mL sheared salmon sperm ssDNA (BioVision, B1677)) was blotted again on the same strip and aloud to air dried. The strip was then transferred into a 1.7 mL Eppdorf tube and blocked with casein blocking buffer at room temperature for one hour. TotalSeq-A antibodies used in this assay were purchased form BioLegend Inc. as follow, TotalSeq-A0132 anti-human EGFR Antibody (352923); TotalSeq-A0404 anti-human CD63 Antibody (353035); TotalSeq-A0373 anti-human CD81 (TAPA-1) Antibody (349521); TotalSeq-A0090 Mouse IgG1, κ isotype Ctrl Antibody (400199). A TotalSeq-A antibody pool was added at concentration of 1:2000 dilution in 100 µL casein blocking buffer and the sample was incubated at 4 °C overnight. On the following day, strips were washed 5 times with PBS-T (0.05% tween-20 in PBS) and one more time with ddH_2_O. Excess liquid was removed using absorbing paper and strip was transferred into a new PCR tube. Component of 1X buffer 2 (NEB, B7002S), 1 U Klenow enzyme (NEB, M0212), dNTP (NEB, N0447, working conc. 20 µM), and synthesized 3′-Adaptor (working conc. 500 nM) were mixed as the extension mix on ice. Fifteen microliters extension mix was added into PCR tube to fully submerge the strip. The tube was then incubated at room temperature for 5 min and heat-inactivated at 95 °C on a PCR cycler (Eppendorf, UX-93944-39) for 5 min. The supernatant now was ready for use as qPCR template of all the TotalSeq antibodies tested. The full length TotalSeq DNA products were quantified by universal-R primer and TotalSeq forward primer (Table 4) in a 15 µL qPCR reaction.

### Protocol of using TotalSeq-A antibody on cells

The protocol was modified from routinely used staining protocol for flow cytometry experiment and BioLegend suggested protocol for CITE-Seq experiment with TotalSeq antibody (https://www.biolegend.com/en-us/TotalSeq).

Cells were dissociated with PBS-EDTA (5 mM) and diluted to 1.0 × 10^6^/100 µL with cell staining buffer (1.0% casein in PBS, 0.02% tween-20, 100 µg/mL sheared salmon sperm ssDNA, 1 µL Fc blocker (BioLegend, 422302)). TotalSeq-A antibody was incubated with cells at 4 °C for 30 min. Cells were washed with cell staining buffer for a total of 3 times. After the final wash step, the cell pellet was resuspended in 30 µL ddH_2_O and lysised by three freeze-and-thaw cycles on dry ice. The tube was then heated to 95 °C for 10 min to further release oligos of TotalSeq antibodies and RNAs within the cells. After a brief centrifuge, cleared supernatant was transferred into a new PCR tube for the extension reaction as described above, but ddH_2_O was replaced by the cleared supernatant. The cleared supernatant was also used for regular RNA isolation, reverse transcription and qPCR quantitation of interested genes.

### RNA isolation, reverse transcription and quantitative PCR

Total RNA from cell, EV or cleared supernatant were isolated by Trizol reagent (ThermoFisher, 15596026) following manufactory’s protocol. Purified RNA sample were dissolved in PCR grade nuclease free water, and quantified by Nanodrop. Up to 1 µg of total RNA were converted into cDNA by qScript cDNA SuperMix (QuantBio, 95048) in 20 µL reaction. One microliter of the cDNA was used as template in 15 µL qPCR reaction containing 1X PerfeCTa SYBR Green FastMix (QuantaBio, 95072) and gene specific primer (250 nM final conc. Table 5). The protocol for either regular gene test or quantification from TotalSeq oligos was optimized and includes; 95 °C for 5 min, 40 cycles of 95 °C for 10 s, 60 °C for 10 s, 72 °C for 20 s, followed by a melt curve assay to prove specific product in the reaction. The qPCR was carried out on CFX Connect Real-Time PCR Detection System (Bio-Rad) and data were analyzed by CFX Manager Software (Bio-Rad) and Excel with ΔΔCt method.

**Table 5:** Sequence of other qPCR primers.

Gene symbol (Alias)	Sequence	Reference or Primer Bank ID^54^
GAPDH	GGAGCGAGATCCCTCCAAAAT	378404907c1
	GGCTGTTGTCATACTTCTCATGG	
ACTB	TCACCCACACTGTGCCCATCTACG	^55^
	CAGCGGAACCGCTCATTGCCAATG	
CD63	CAGTGGTCATCATCGCAGTG	91199544c1
	ATCGAAGCAGTGTGGTTGTTT	
CD9	TTCCTCTTGGTGATATTCGCCA	319738657c2
	AGTTCAACGCATAGTGGATGG	
PDCD6IP (Alix)	ATCGCTGCTAAACATTACCAGTT	371875333c2
	AGGGTCCCAACAGTATCTGGA	
SDCBP (Syntenin-1)	TGGCTCCTGTAACTGGTAATGA	55749522c2
	CTCAGACCAACCAATGAGGCT	
SRSF5 (HRS)	AACGACAAGAACCCACACGTC	315138978c2
	GGCCTGGATCAGGTACAGGA	
TSG101	ATGGCTACTGGACACATACCC	332000018c2
	GCGGATAGGATGCCGAAATAG	
CHMP1A	GTGTATGCCGAGAACGCCAT	379139237c1
	TTGGAGGCCACTGCGTCTA	
CHMP2A	CGCGAGCGACAGAAACTAGAG	38372936c1
	CCCGCATCAATACAAACTTGC	
CHMP4C	ACTCAGATTGATGGCACACTTTC	62526041c3
	GCTGCAAAGCCCATGTTCC	
CHMP5	GACACCAAGACCACGGTTGAT	306966145c2
	GGGTGCCATAACTGCGACTC	
ITGA2	GGGAATCAGTATTACACAACGGG	116295257c2
	CCACAACATCTATGAGGGAAGGG	
ITGA3	TGTGGCTTGGAGTGACTGTG	171846264c2
	TCATTGCCTCGCACGTAGC	
ITGB1	CAAGAGAGCTGAAGACTATCCCA	182507160c3
	TGAAGTCCGAAGTAATCCTCCT	
ITGB2	TGCGTCCTCTCTCAGGAGTG	188595673c1
	GGTCCATGATGTCGTCAGCC	

### Flow cytometry

HEK293, HEK293-CD63 and HEK293-CD63-CRISPR cells were dissociated from cell culture flask by PBS-EDTA. Then cells were washed with PBS and blocked with flow cell staining buffer (PBS, 1% BSA, Fc Blocker) at 4 °C for 5 min. CD63-PE (BD Bioscience, 557305) or matched isotype control (BD Bioscience, 556650) antibody was incubated with cells for 30 min, and then washed 3 times with staining buffer. The stained cell samples were run on FACSCanto flow cytometry system (BD Bioscience) cytometer in FSU College of Medicine Core Facility, and CD63 expression level was quantified by geometric mean value on FACSDiva software (BD Bioscience).

### Western blot

Cell lysates were prepared by directly adding RIPA buffer with proteinase inhibitor cocktail (ThermoFisher, 78429) on to the cell culture plate and incubated on ice for 10 min. Then all the lysate was transferred into a clean Eppendorf tube and centrifuged at 10,000 g for 15 min. Cleared supernatant was stored in − 80 °C until use. EV samples were lysed by directly mixing with 5X SDS-PAGE sample buffer and boiled. Protein quantification was measured by 660 nm Protein Assay. Ten to twenty micrograms of protein from cell or EV samples were loaded into 10% sodium dodecyl sulfate and polyacrylamide gel (SDS-PAGE). For immunoblotting, proteins were transferred to a nitrocellulose membrane (GE Healthcare, #10600002). The membranes were blocked in a standard TBS-T buffer with 5% non-fat dry milk for one hour at room temperature. Membranes were then probed for EV markers of CD63 (ThermoFisher, 10628D), CD81 (GeneTex, GTX72476), CD9 (Abcam, ab2215), TSG101 (GeneTex, GTX70255), and EGFR (SantaCruz, sc-03) by overnight incubation at 4 °C. These primary antibodies were subsequently probed with HRP conjugated anti-mouse IgG (GeneTex, GTX213112) or anti-rabbit IgG (GeneTex, GTX77060). ECL substrate was added for picogram (Azure, 10147-296) protein detection thresholds. Chemiluminescence signal was detected using the LAS4000 luminescent image analyzer (GE Healthcare Life Sciences) and software Version 8.1 of Image Quant-TL (GE Healthcare Life Sciences).

### Micro size exclusion chromatography (micro-SEC)

For the micro-SEC, 500 µL Sepharose CL-2B (Sigma, CL2B300) was packed into Micro Bio-Spin Chromatography Columns (Bio-Rad, 7326204) by gravity flow. Particle-free PBS was used as mobile phase and fractions were collected dropwise. For comparison, 10 mL bed volume column was packed in syringe as described in the literature^26^.

One milliliter of pre-cleared human serum sample was fractionated on regular size column, and EV fraction was considered separated in 3 to 6 mL based on reference^26^. Ten or twenty-five microliter of pre-cleared sample were used for micro-SEC and fractions were collected to test CD63 levels, protein and EV concentration using the method or instrument described above. Equal volume of fractions was loaded into SDS-PAGE gels to analyze EV markers such as CD63, CD81, EGFR and TSG101 by western blot. The same samples lysed with SDS were also used for TotalSeq assays.

### CD63 direct ELISA

Direct ELISA method was used to show EV enrichment in micro-SEC method. Briefly, fractions were coating on the MaxiSorp plate (NUNC, 44-2404-21) by diluted in 100 µL bicarbonate/carbonate buffer overnight at 4 °C. After blocking with PBS + 2%BSA at room temperature for one hour, CD63 antibody (ThermoFisher, 10628D) were added to each well at 1:250 dilution and incubated overnight again to maximize performance. Anti-mouse IgG HRP (GeneTex, GTX213112) was used at 1:1000 concentration as secondary antibody. Between each step the plate was washed 5 times with at least 300 µL PBS-T to remove any unbound antibody. One hundred microliters of TMB substrate solution (BioLegend, 421101) was developed for 10 min in dark or until it reached the desired color. The reaction was stopped with 50 µL 2 N sulfuric acid and signals were read at 450 nm.

### Statistical analysis

Raw data were collected from the software used with instrument of plate reader, gel imager or flow cytometer. Experiments were carried out at least in two to three biological replicates, and data is represented as a mean + standard deviation. Statistical significance was determined by student-t test with *p* < 0.05. Excel software were used for column and scatter chart.

## Supplementary information

Supplementary Information.
