# Supplementary material for: Multiplex protein profiling method for extracellular vesicle protein detection
Source: Sci Rep. 2021 Jun 14;11:12477. doi: 10.1038/s41598-021-92012-6 (PMC8203679; doi:10.1038/s41598-021-92012-6)
Supplement: Supplementary file 1 — Supplementary Information. [file 41598_2021_92012_MOESM1_ESM.pdf]

Supplementary Information

Multiplex protein profiling method for extracellular vesicle protein detection from small sample volumes

Li Sun and David G. Meckes Jr*

Department of Biomedical Sciences, Florida State University College of Medicine, Tallahassee, FL, 32306

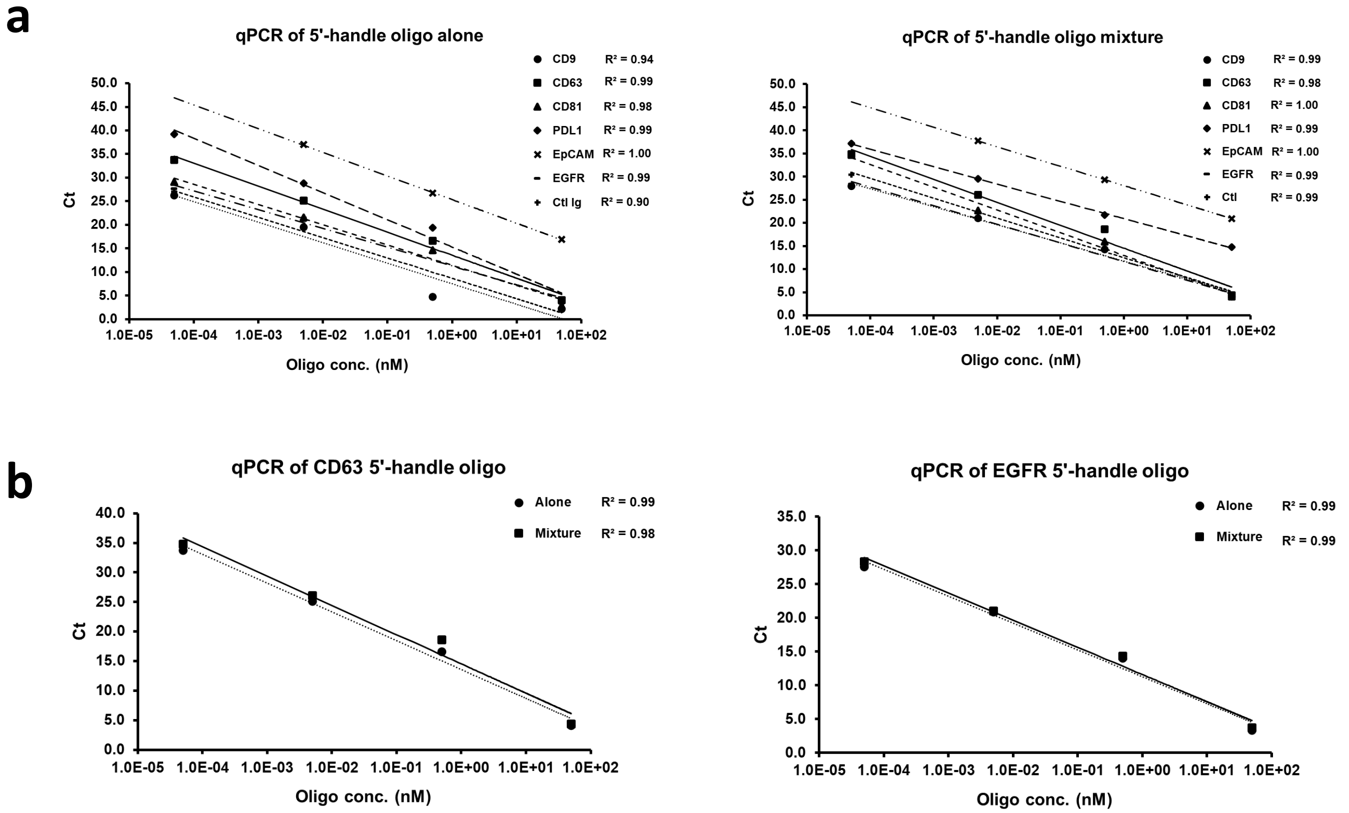

Supplementary Figure S1. qPCR on serial dilution of TotalSeq Oligos. (**a**) Ten-fold dilution of seven different TotalSeq Oligos were performed only the ligation and qPCR step. The oligos were either tested alone (left) or in a mixture (right). (**b**) Two dilution curves from same oligo alone or mixed sample were plotted in the same chart.

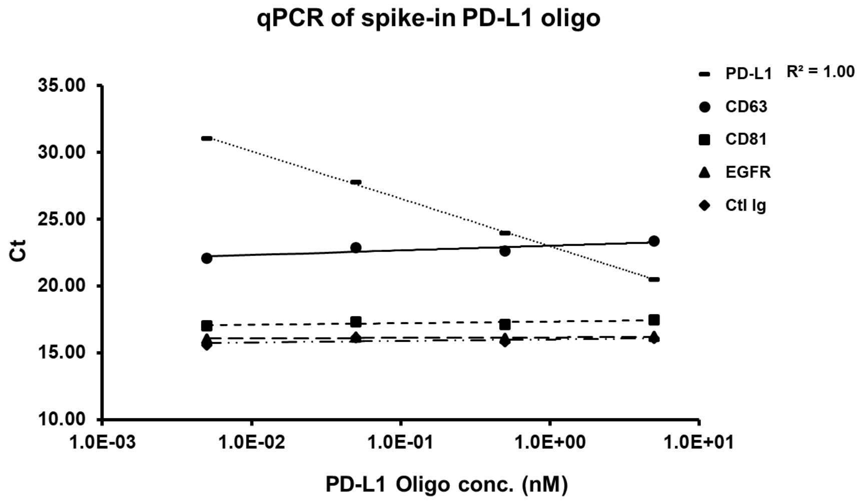

Supplementary Figure S2. Spike-in oligo into other oligo mixture. Different concentration of PD-L1 oligo were spiked into an equal molar mixture of four other oligos (CD63, CD81, EGFR, Ctl Ig).

Supplementary Table S1. qPCR primer specify test

		Oligo Sample						
		**CD9**	**CD63**	**CD81**	**PD-L1**	**EpCAM**	**EGFR**	**Ctl Ig**
**qPCR Primer**	**CD9**	4.73	34.98	35.63	34.56	32.16	36.24	34.55
	**CD63**	N.D.	16.61	N.D.	N.D.	N.D.	N.D.	N.D.
	**CD81**	N.D.	37.81	14.59	N.D.	N.D.	N.D.	N.D.
	**PD-L1**	N.D.	N.D.	N.D.	19.40	N.D.	N.D.	N.D.
	**EpCAM**	N.D.	N.D.	N.D.	N.D.	26.68	N.D.	N.D.
	**EGFR**	N.D.	32.92	37.17	39.19	N.D.	14.00	N.D.
	**Ctl Ig**	N.D.	N.D.	N.D.	N.D.	N.D.	N.D.	4.80

0.5 nM of each oligo sample was tested with all other qPCR primers, Ct value of qPCR was shown in the table. N.D. = not detectable

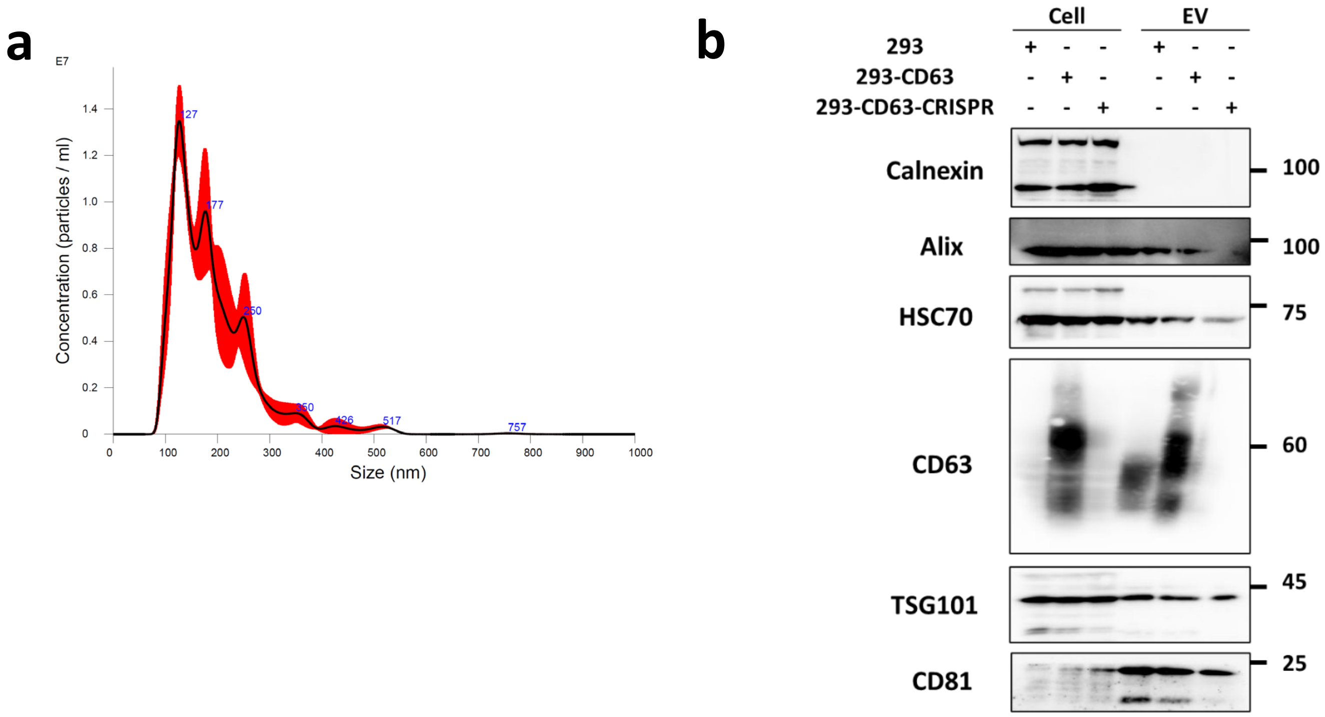

Supplementary Figure S3. Characterization of isolated EVs. (**a**) Size distribution of isolated EVs were measured by NTA. (**b**) Western blot results of isolated EV and corresponding cell lines. Full-length blots are presented in Supplementary Figure S9.

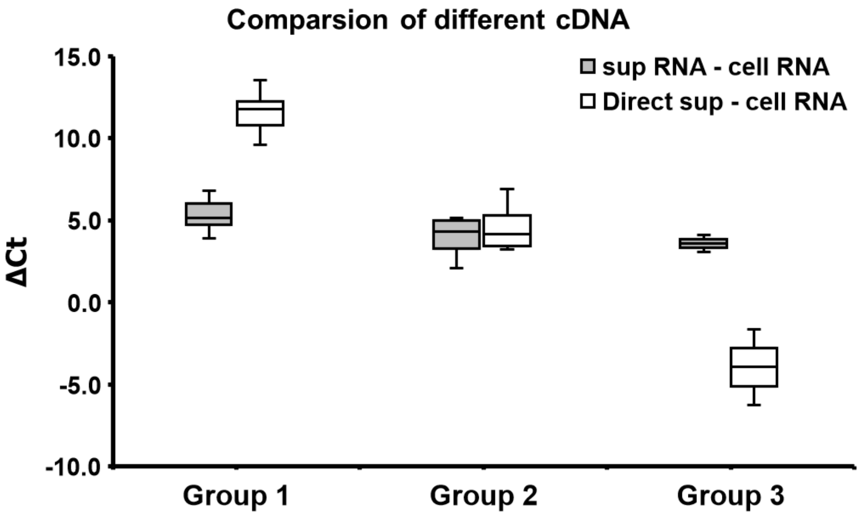

Supplementary Figure S4. Ct value comparison of cDNA from isolated RNA or direct reverse transcription. Average Ct values of RNAs isolated from cell, RNAs isolated from supernatant and supernatant directly into cDNA were clustered into 3 groups. ΔCt was the difference compared with RNAs isolated from cell.

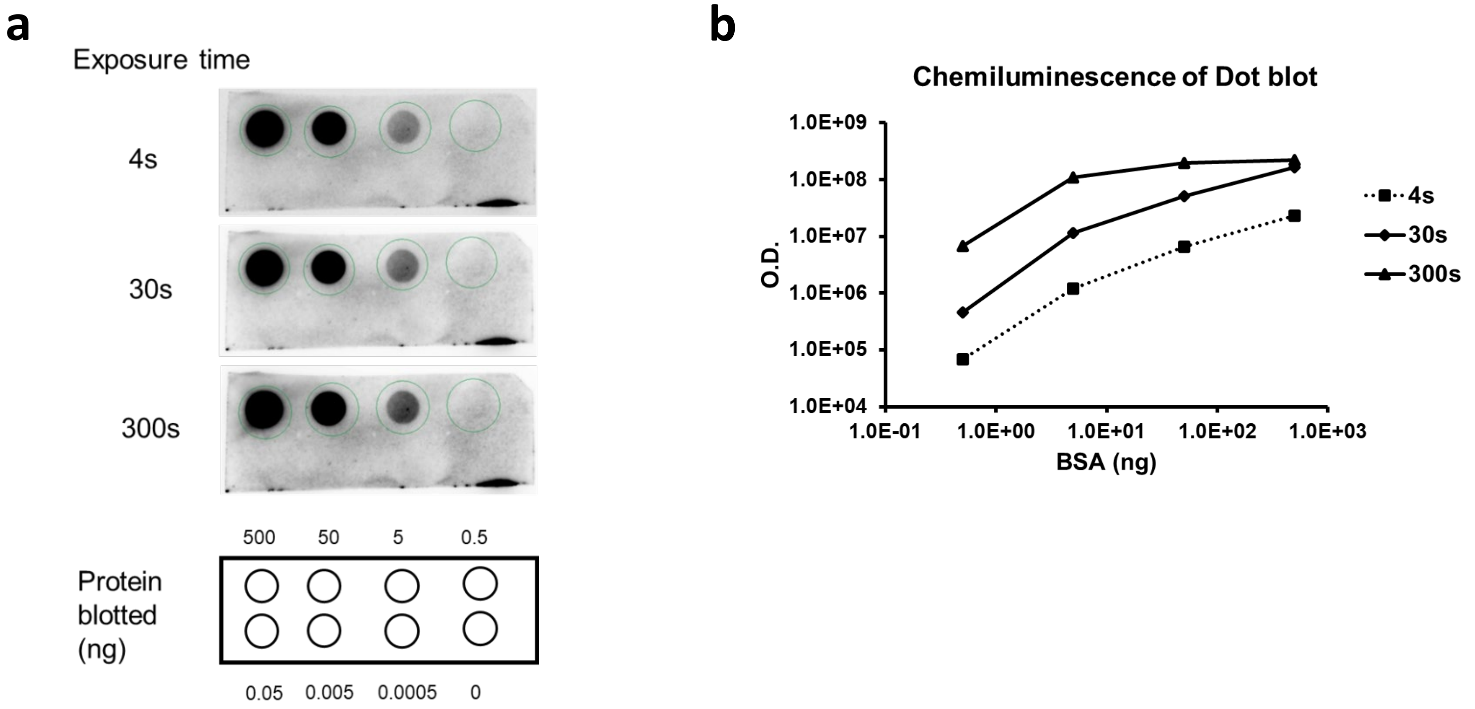

Supplementary Figure S5. Dot blot of BSA. (**a**) Serial dilution of biotinylated BSA were blotted on the NC membrane and tested under standard protocol with streptavidin-HRP. The membrane was exposed for 4s, 30s or 5 minutes. (**b**) The plot shows optical intensity to protein amount.

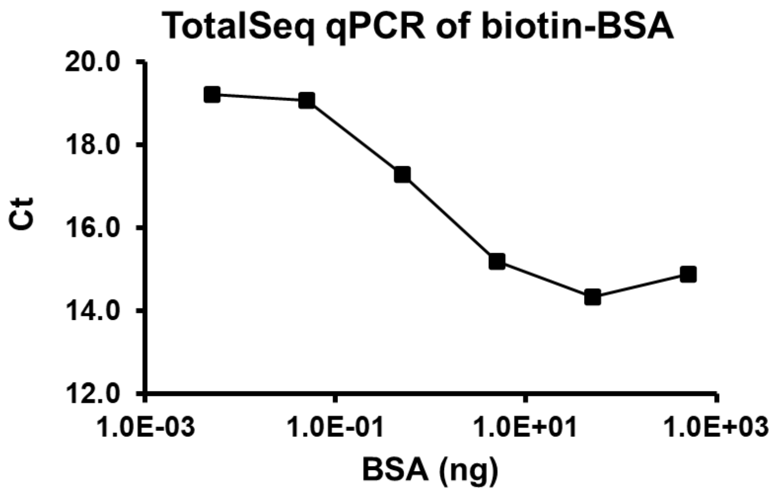

Supplementary Figure S6. TotalSeq Protocol test with Biotin-BSA and Streptavidin-Oligo. Biotinylated BSA was blotted on the strip as analyte, and probe by oligo conjugated streptavidin. Ct of qPCR was plotted to BSA amount.

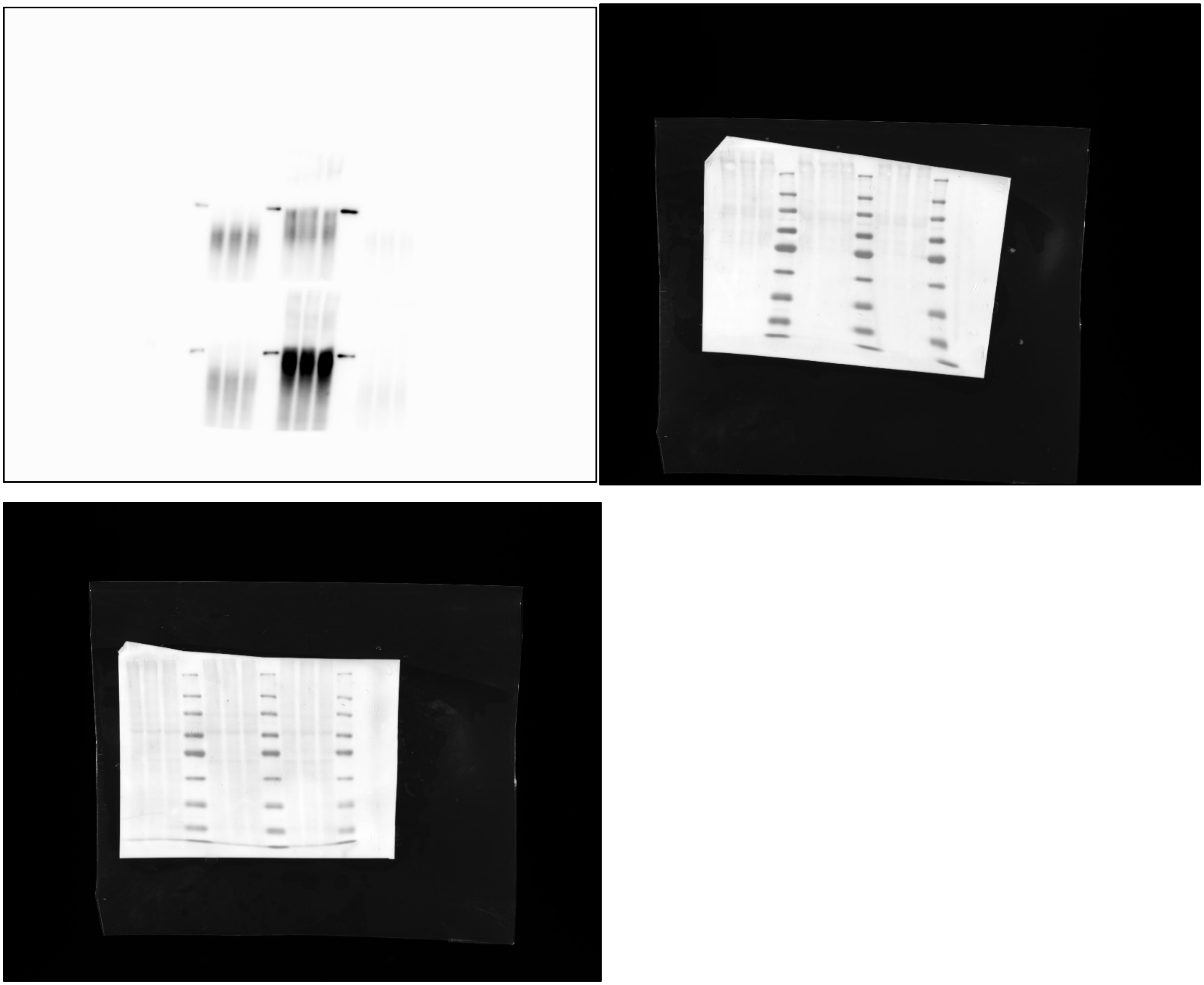

Supplementary Figure S7. Original western blot images of Figure 1e.

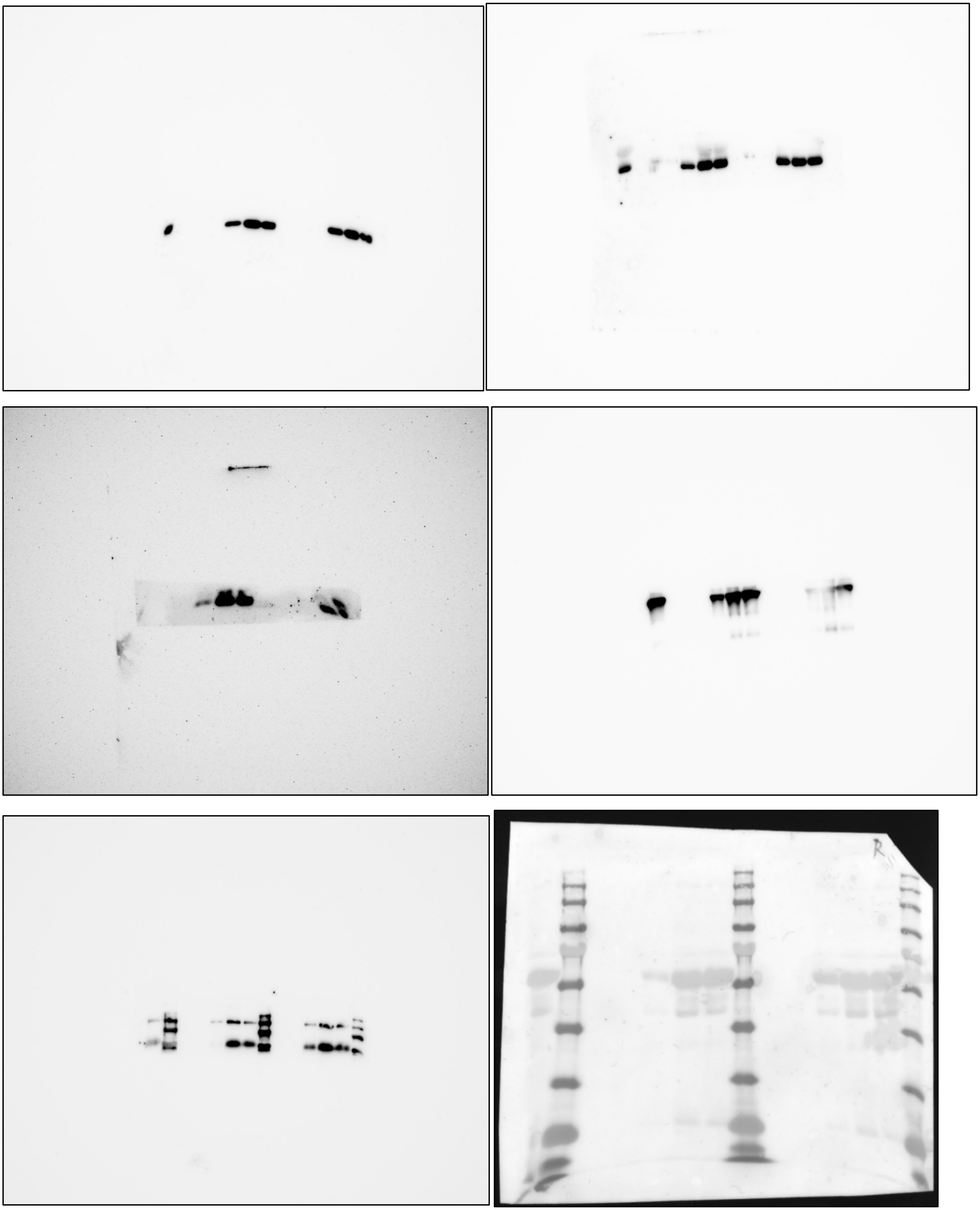

Supplementary Figure S8. Original western blot images of Figure 4b.

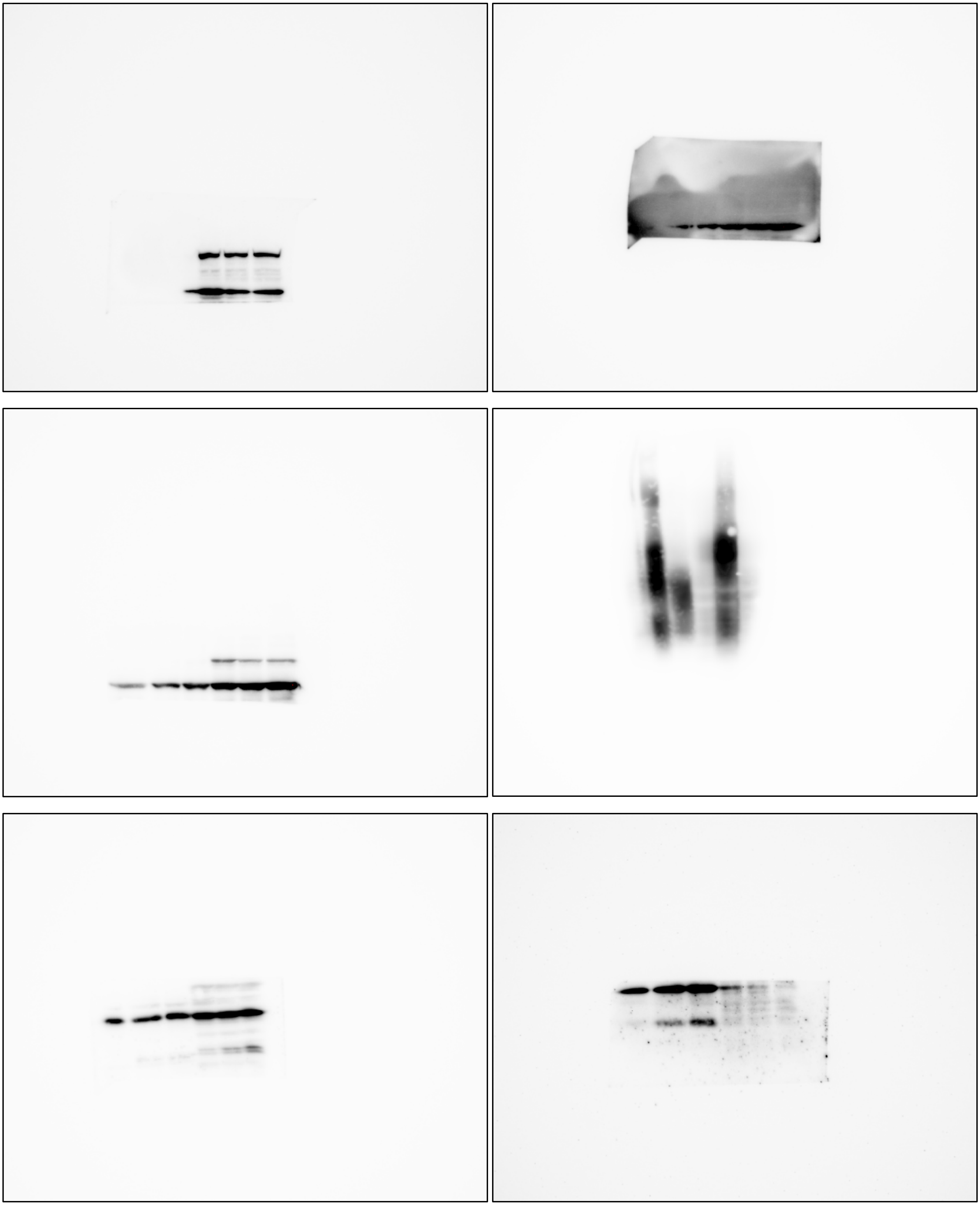

Supplementary Figure S9. Original western blot images of Supplementary Figure S3.
